# How reliable is the labeling of a commercial phytosteroid product? A 12-week randomized double-blind training study

**DOI:** 10.1080/15502783.2025.2540408

**Published:** 2025-08-08

**Authors:** Joshua Dissemond, Tim Havers, Steffen Held, Stephan Geisler, Tihomir Kostov, Patrick Diel, Svea Türschmann, Maria K. Parr, Eduard Isenmann

**Affiliations:** a IST University of Applied Sciences, Department of Fitness and Health, Duesseldorf, Germany; b Technical University of Munich, Department Health and Sport Sciences, TUM School of Medicine and Health, Munich, Germany; c IST University of Applied Sciences, Department of Sport and Management, Duesseldorf, Germany; d German Sports University Cologne, Department of Molecular and Cellular Sports Medicine, Institute for Cardiovascular Research and Sports Medicine, Cologne, Germany; e Freie Universitaet Berlin, Institute of Pharmacy, Pharmaceutical and Medicinal Chemistry (Pharmaceutical Analysis), Berlin, Germany

**Keywords:** Phytosteroids, ecdysterone, diosgenin, hypertrophy, resistance training

## Abstract

**Introduction:**

Phytosteroids like 20-hydroxyecdysone (20E) and diosgenin (DSG) have shown promising anabolic and performance-enhancing effects in in vitro, animal, and human studies. Combining phytosteroids is common in supplements, with early in vitro research suggesting additive effects via distinct signaling pathways. However, human studies on the combined effects of 20E and DSG are lacking. This study aimed to evaluate the anabolic and performance-enhancing potential of a commercially available 20E and DSG supplement.

**Methods:**

Twenty-eight resistance-trained young men were recruited for this study. Participants were randomized into two groups: a 20E and DSG (EcDi) group, and a placebo (Plac) group. Both groups performed free-weight resistance training three times per week for 12 weeks. The EcDi group received a commercially available phytosteroid product three times a day (as recommended on the label), while the Plac group received a placebo product. Assessments were conducted at four time points (T0, T1 [4 weeks], T2 [8 weeks], T3 [12 weeks]) and included measurements of one-repetition maximum (1-RM) for the squat (SQ) and bench press (BP) exercises, as well as body weight (BW), fat-free mass (FFM), muscle mass (MM), fat mass (FM), and muscle thickness of the pectoralis major (PM) and the proximal, middle and distal regions of the anterior (i.e. *M. rectus femoris* and *M. vastus intermedius*) and lateral quadriceps femoris (i.e. *M. vastus lateralis* and *M. vastus intermedius*). Liver and kidney function, along with endocrine parameters, were measured via blood samples pre- and post-intervention. Additionally, the concentration of 20E and DSG in the product was measured by LC-MS/MS, and its biological activity was evaluated using C2C12 cells after the intervention. Data were analyzed using a linear mixed model (LMM).

**Results:**

Twenty-four participants completed the study successfully. Significant improvements in 1-RM SQ and BP were observed across both groups, indicating a time but no specific group effect. Similarly, significant time effects without group effects were found for FFM, MM, FM and muscle thickness (anterior quadriceps femoris at 30%, lateral quadriceps femoris at 50% and PM). Subsequent analyses of the supplement revealed that less than 1% of the claimed 20E concentration and 10.4% of the claimed DSG concentration were present in the capsules. In addition, no biological activity or hypertrophic effects were detected in the C2C12 cells.

**Conclusion:**

This study demonstrated that the prescribed resistance training protocol resulted in significant anabolic and performance-enhancing effects that did not differ between groups. The lack of group-specific differences suggests that the tested phytosteroid supplement did not provide additional benefits. The significant discrepancy between the claimed and actual 20E and DSG concentration resulted in the lack of biological activity in C2C12 cells and likely contributed to the absence of measurable group-specific effects. To enhance the reliability of future phytosteroid research, we strongly recommend: (1) verification of active compound concentrations in supplements, its actual content and absence of potential contamination with prohibited substances, (2) confirmation of their biological activity using *in vitro* models. Implementing these measures will help to minimize inconsistencies and enhance the reliability of future studies on phytosteroids.

## 1. Introduction

The use of dietary supplements is common in sports and varies depending on the specific sports discipline [1,2]. One of the main reasons for athletes to take nutritional supplements is to enhance their athletic performance, as even minor differences in performance can be decisive for success [3–6]. In this context, dietary supplements play an important role in athletes’ recovery, training adaptation, and performance.

Phytosteroids, like 20-hydroxyecdysone (20E) or diosgenin (DSG) are gaining attention for their potential performance-enhancing and anabolic effects [7–10]. Ecdysteroids are a class of polyhydroxylated steroids found in plants and other organisms, with 20E being the most abundant and biologically active form of ecdysteroids in insects [11,12]. Research on the effects of 20E in mammals shows promising results, indicating anabolic, fat-reducing, anti-diabetic, anti-inflammatory, and cardio-, bone-, and cartilage-protective properties [11]. However, the exact mechanism of action of 20E in mammals remains unclear, though *in vitro* studies suggest it may operate through various pathways, including a membrane G-protein coupled receptor (GPCR) and nuclear estrogen receptor-β (ERβ), with recent research proposing a combined interaction involving the GPCR Mas receptor and a membrane-bound estrogen receptor [10–15]. In addition recent studies demonstrated anabolic effects *in vitro* and in animal models [16–21]. Furthermore investigations in mice and rats aimed to identify metabolites to better understand the pharmacological effects of 20E [22,23].

Current studies in humans, however, are rather inconclusive. Isenmann et al. and Pérez-Piñero et al. were able to observe both performance-enhancing and anabolic effects through 20E application [7,24], whereas Willborn et al. were unable to detect any effect [25]. In comparison to the study by Wilborn et al., the actual 20E content was examined in the two other studies, but only Isenmann et al. also reported testing for the presence or absence of other prohibited substances and for anabolic effects in cell culture [7,24]. Isenmann et al. reported a total of 6 mg of 20E per capsule, corresponding to a daily dosage of 12 mg (84 mg/week) in the low-dose group and 48 mg (336 mg/week) in the high-dose group [7]. In contrast, the total daily dose in the study by Pérez-Piñero et al. was only 3.2 mg (22.4 mg/week) [24]. In addition to studies involving healthy and trained individuals, 20E is currently being investigated as a phytopharmaceutical in preliminary clinical pilot trials (phases 1 and 2). Single-dose administrations of 175 mg and 350 mg were well tolerated, with no significant adverse effects observed [26]. Furthermore, emerging data indicate that 20E can significantly enhance muscle function in geriatric populations and may reduce the risk of mortality and respiratory failure following COVID-19 infection [27,28].

Furthermore, Ambrosio et al. compared various 20E supplements and found that most products did not contain the amount of 20E stated on their labels [29]. Only three of the 12 analyzed supplements contained 20E levels that complied with the allowable deviation of 50% between the labeled and actual content [30]. Two of these products labeled to contain suma root reported 20E concentrations ranging from 1.7 to 3.3 mg/capsule (measured concentration 1.37 to 2.32 mg/capsule), while one product using spinach extract declared a 20E content of 25 mg/capsule (measured concentration 22.29 mg/capsule) [29]. These findings of Ambrosio et al. highlight the lack of quality assurance in supplement formulations and the inconsistency in human intervention studies. In addition to widely varying ingredient contents, dietary supplements may also be contaminated with banned substances. It is estimated that 6.4–8.8% of positive doping cases can be attributed to the use of contaminated dietary supplements [31–33]. Even worse, recent studies have shown that nearly 20% of tested 20E supplements contained impurities, including prohibited anabolic substances [34]. Therefore, if 20E and contaminations are not specifically analyzed, any observed effects could potentially be related to contamination rather than the compound itself.

Based on the effects in cell culture, animal models, and partly also in human studies, the World Anti-Doping Agency (WADA) has included 20E in their monitoring program in 2020 for their anabolic and performance-enhancement effects [35].

In addition to supplements, 20E can also be ingested through food sources. Spinach and quinoa in particular contain relatively large amounts of 20E [22,36]. However, the concentrations in food varies, depending on variety, genetics and environmental conditions [36,37]. The 20E content in quinoa seeds ranges from 70 to 800 μg/g and in spinach ranges from 4 to 800 μg/g fresh weight and 17 to 885 μg/g dry weight [22,36–38].

Recent studies have shown that, despite the consumption of large amounts of spinach ( > 900 g; 20 ± 3 µg/g 20E, approximately 18–19 mg/serving) and quinoa (150 g raw; 370 ± 14 µg/g 20E, approximately 53.3 mg/ serving), only 1–3% of 20E and its metabolites are absorbed. In contrast, administration of 50 mg of pure 20E resulted in approximately 50% recovery in urine, indicating significantly higher bioavailability compared 20E from food sources [39–41].

Following the promising effects of 20E, the phytosteroid DSG, found in fenugreek and yams, has also attracted considerable attention for its potential therapeutic and performance-enhancing properties [42]. DSG is of high interest for scientific research due to its postulated pharmacological properties against certain diseases such as diabetes, hyperlipidemia, cancer, cardiovascular diseases, metabolic syndrome, oxidative stress and inflammation [42–46]. Like 20E, DSG reportedly interacts with several signaling pathways, including NF-κB, JAK/STAT, and PI3K/AKT/mTOR as well as MAPK and several others [45]. Beyond its pharmaceutical applications, DSG has been studied for its effects on performance, body composition, and hormones. A meta-analysis by Isenmann et al. found small effects in lower body performance, body composition, and blood testosterone levels in healthy and trained men [8]. A small reduction in fat mass (FM) and estradiol concentration (E2) were also detected. However, the small sample size (*n* = 378) and limited number of studies (k = 6) suggest caution in interpreting these results. Similarly, a study by Rao et al. showed that high-dose fenugreek (600 mg Libifem) increased leg press strength and lean mass while reducing fat mass compared to placebo in women [47]. These effects were dose-dependent and similar to previous findings in male participants [8].

Apart from the individual anabolic effects of the two substances, there are already initial recommendations to combine phytosteroids such as 20E and DSG [48]. Combinations of 20E and DSG have shown promising results and are the subjects of ongoing research. Kostov et al. [21] investigated the combined effect of 20E and DSG in C2C12 cells at different dosages and showed significantly higher hypertrophic effects in three of the five combinations compared to the individual substances [21]. To our knowledge, the potential anabolic and performance-enhancing effects of the combination of 20E and DSG have not yet been investigated in humans. Therefore, the aim of this study was to examine changes in 1-RM performance, body composition, muscle thickness and several biomarkers in response to a 12-week free-weight-based resistance training (RT) program in strength-trained male athletes using a commercially available phytosteroid supplement compared to placebo. It was hypothesized that participants taking the combined supplement of 20E and DSG would have higher increases in 1-RM performance, muscle thickness, and better results in body composition and several biomarkers compared to placebo. The results of this study will provide additional data to improve the understanding of the currently contradictory findings regarding phytosteroid supplementation and its effects on performance enhancement in male athletes.

## 2. Material and methods

### 2.1. Participants and ethical approval

**Figure 1.**
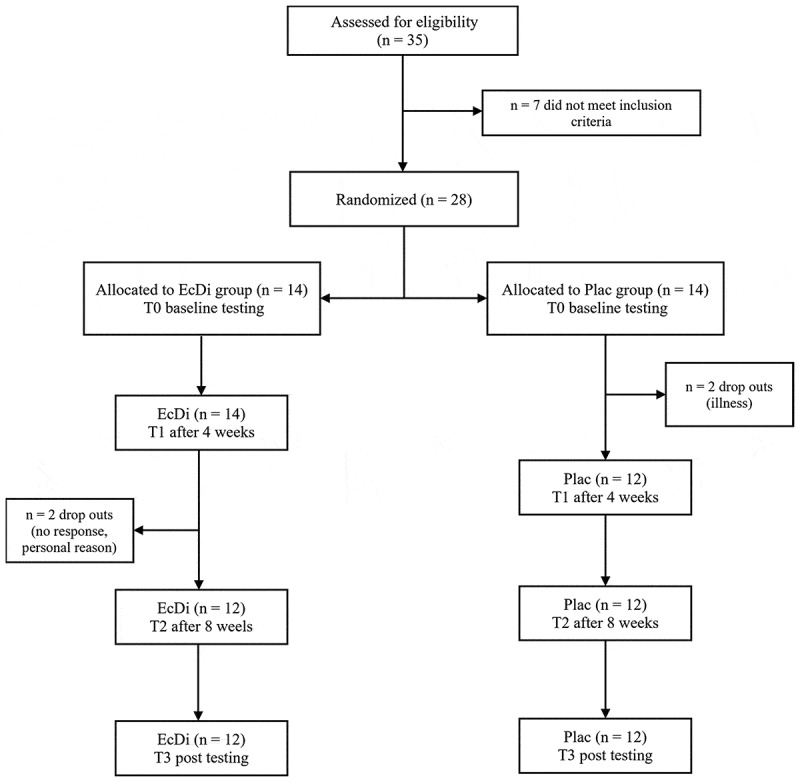
Participant flow diagram. EcDi, ecdysterone + diosgenin group; Plac, placebo group.

To determine the required sample size, an *a priori* power analysis was conducted using F-tests (ANOVA: fixed effects, special, main effects, and interactions). The calculation was based on a medium effect size (f = 0.25), with an α-error of 0.05 and a power of 0.8 (1-β error), according to a previous study [7]. For a two-arm design (df = 1) and four measurement time points, the total sample size was 24 participants. However, considering the 12-week study duration and the potential for dropouts, the minimum sample size was adjusted to 28 participants. Initially, 35 male athletes were recruited, out of which 28 met the inclusion criteria. Twenty-four subjects completed the study and were included in the analysis. Two of the subjects were lost due to illness, one dropped out for personal reasons and one participant did not respond after the second test session. Figure 1 outlines subject recruitment, randomization, and reasons for dropout.

All volunteers were recruited at a local gym (Windhagen, Germany) and met the following criteria: 1) at least 18 years old, 2) free from neuromuscular/musculoskeletal disorders and known chronic diseases (cardiovascular disease, type-2 diabetes mellitus, etc.), 3) no consumption of any type of phytosteroid supplements in the last 6 months, 4) no consumption of anabolic steroids, 5) 1-RM SQ of 120% body weight (BW) and 1-RM BP of 100% BW or higher, 6) at least 3 years of RT experience with a minimum of 3 sessions per week; 7) were nonsmokers. According to Santos et al. [49] and McKay et al. [50], the inclusion criteria for 1-RM and RT experience ensure that all subjects are at least advanced, well-trained athletes. The investigation was approved by the local ethics committee of the IST University of Applied Sciences, Düsseldorf (Nr.06/2023) and registered in the German register for clinical trial (DRKS00036224). Before the subjects were included in the study, they were informed about the study design and objectives and had to give their written consent to participate. All personal data collected was anonymized and complies with the data protection regulations in Germany.

### 2.2. Experimental design

**Figure 2.**
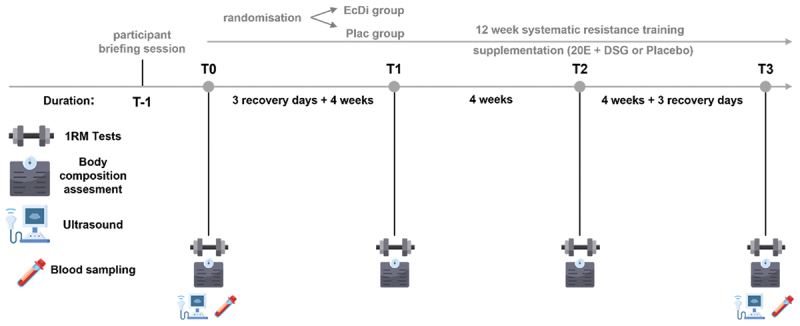
Experimental design. EcDi, ecdysterone + diosgenin group; Plac, placebo group; 1-RM, one-repetition-maximum; 20E, ecdysterone; DSG, diosgenin; *T*-1, initial visit; T0-T3, measurement times.

A randomized, placebo-controlled, double-blind study was used to investigate the effects of a commercially available supplement on 1-RM performance, body composition, muscle thickness, endocrine system, liver and kidney function. Figure 2 illustrates the experimental design of the study. At the initial visit (*T*-1), all individuals interested in participating in the study attended a participant briefing session outlining the study purpose and providing all necessary details for participation. The study consisted of a 12-week intervention with four measurement points (T0, T1, T2, T3) and a 3-day recovery period after the baseline test at T0 and before the posttest at T3.

At T0 and T3, all athletes were assessed for body composition, hormonal status, kidney and liver function, muscle thickness, and dynamic strength. The testing session at four (T1) and eight weeks (T2) of the intervention consisted of body composition and 1-RM assessments only. After baseline testing, subjects were randomly assigned to either the ecdysterone and diosgenin group (EcDi) or the placebo group (Plac) using stratified randomization based on age and relative strength in squat (SQ) and bench press (BP). Six strata were created based on three levels of relative strength for squat (SQ: < 1.25; 1.26–1.49; ≥1.50) and bench press (BP: < 1.05; 1.06–1.30; > 1.30), as well as three age groups (19–25, 27–34, ≥35). After stratification, each of the six strata was randomized into two groups using a randomizer (ultimatesolver.com). The 20E + DSG and the placebo supplements were then administered to the participants after the 3-day recovery period.

### 2.3. Procedures

Participants were not allowed to conduct RT or any other strenuous physical activity for 48 hours before testing. In addition, the participants had to arrive sober and were not allowed to consume any soft drinks, coffee or alcohol. However, the volunteers were instructed to drink 400–500 ml of water in the morning before testing to ensure that their water balance was equalized. The order of measurements at T0 and T3 was as follows: 1) blood samples, 2) body composition, 3) muscle thickness, and finally 1-RM SQ and BP. At T1 and T2, only body composition and 1-RM SQ and BP were measured, in this order. After the muscle thickness assessment and before the 1-RM tests, the participants consumed 1 g of oats per kilogram of body weight, one banana, 20 g honey, and hot water *ad libitum* to ensure sufficient energy availability. At T1 and T2, the meal was taken between the body composition assessment and the 1-RM tests. After the meal, participants had 30–60 minutes before the 1-RM assessment began. All measurements (T0-T3) were conducted in the morning (7:00–12:00 am) at the same time of the day by the same researchers, doctor and volunteer.

### 2.4. Blood samples

To investigate the potential effects of EcDi supplementation on hormone status and metabolism, the following hormonal parameters were analyzed: estradiol (E2), total testosterone (T), free testosterone (fT), cortisol (CORT), and insulin-like growth factor 1 (IGF1). Biomarkers associated with kidney and liver function, which serve as essential indicators of metabolic health, were analyzed. These included creatinine, glutamate-oxaloacetate transaminase (GOT), glutamate-pyruvate transaminase (GPT), and gamma-glutamyl transferase (GGT). Blood samples were collected immediately at the beginning of T0 and T3 (7:00–8:30 am). While the participant was sitting or lying down, a physician applied a tourniquet and used a winged infusion set to venipuncture the participant’s right arm and collect fresh venous blood in a vacutainer. After all blood samples were collected, they were taken to an external laboratory for analysis (Labor Dr. Wisplinghoff, Horbeller Str. 18–20, 50858 Köln, Germany). This laboratory has been used in previous studies [7,51].

### 2.5. Body weight and body composition assessment

All anthropometric measurements were assessed at each time point (T0, T1, T2, T3). For BW assessment, participants wore only underwear and no shoes or socks. A digital scale (Etekcity, EB4074S, Anaheim, California 92,806, USA) was used to assess BW of the subjects. Because the study was conducted in winter, participants were allowed to wear light clothing for the body composition assessment, but were required to remove all forms of jewelry, watches, or any object consisting of metal. Fat-free mass (FFM), MM, FM, total body water (TBW) and body fat percentage (BF%) were analyzed by bioelectrical impedance analysis (BIA 101 AKERN, Florence, Italy). The BIA 101 Akern is a valid and reliable alternative to dual-energy X-ray absorptiometry (DXA) for the assessment of body composition and has been used in various other studies [7,52–54]. Whole-body resistance (R) and reactance (Xc) were measured using an alternating sinusoidal electric current of 400 µA at an operating frequency of 50 kHz. The device was calibrated every morning using the standard control circuit supplied by the manufacturer with a known impedance (resistance (R) = 383 ohm; reactance (Xc) = 45 ohm). The accuracy of the device was 1% for R and 2% for Xc. For the bioelectrical impedance measurement, participants were placed in a supine position with their limbs slightly apart from the body and remained in this position for 10 minutes to allow for fluid shifts. Disposable tab electrodes (BIATRODES, Akern Srl, Florence, Italy) were then applied to the metacarpal and metatarsal sites of the right wrist and ankle [53]. Subsequently, the measurement was performed and the data analyzed using BodyGramPro software (Version 3.0, Akern, Florence, Italy).

### 2.6. Muscle thickness assessment

Muscle thickness was assessed using noninvasive skeletal muscle ultrasound imaging of the right thigh and chest locations of all participants. Measurements were conducted at T0 (baseline) and T3 (post-intervention). A B-mode ultrasound device (Mindray DP-50, Mindray Medical International Ltd, Shenzhen, China) with an 8.5-MHz linear probe (Mindray 75L53EA, Mindray Medical International Ltd, Shenzhen, China) was used, following established procedures from previous studies [55–58]. Prior to image acquisition, all anatomical locations of interest were identified using standardized landmarks. Muscle thickness was assessed for the lateral quadriceps muscle (*M. vastus lateralis* (VL) and *M. vastus intermedius (VI)*), anterior quadriceps muscle (*M. rectus femoris* (RF)and *VI*), and M. pectoralis major (PM). To ensure consistent positioning for repeated measurements, all sites were marked with a waterproof pen. Lateral quadriceps thickness was measured at 30%, 50% and 70% of the distance between the most prominent point of the greater trochanter and the lateral condyle of the tibia (gain 50 dB) with the participants lying on their left side on an examination table. Landmarks of the anterior quadriceps femoris were set at 30%, 50% and 70% of the distance between the anterior inferior supra-iliac crest and the proximal border of the patella. Muscle thickness measurements of the anterior thigh were obtained with the subjects in the supine position (gain 50 dB). In the same supine position, the PM was measured on the right side between the third and fourth rib, below the midpoint of the clavicle (gain: 50 dB). The arms remained relaxed at the sides of the body. Muscle thickness was determined using the borders of the subcutaneous adipose tissue and the muscle-bone interface as reference points [56].

A water-soluble ultrasound transmission gel was applied to the probe to ensure optimal contact. The probe was carefully placed perpendicular to the muscle’s long axis, avoiding any compression of the underlying tissue. For each muscle, three images were captured and stored digitally. Muscle thickness was then analyzed using the caliper measurement tool of the ultrasound device. If one of the images showed a difference of more than 10%, a fourth image was taken and used as a reference instead of the deviating image. The mean value of the three images was used for further analysis. Muscle thickness measures were performed by an experienced and trained ultrasound technician, blinded to group allocation. The three images taken at T0 and T3 were used for further analysis of the coefficient of variation (CV) and the intraclass correlation coefficient (ICC). ICC estimates and their 95% confidence intervals were calculated using R (version 4.4.1) and RStudio, based on a mean-rating (k = 3), absolute-agreement, two-way mixed-effects model (ICC(3,k). The ICC(3,k) and CV values for the lateral quadriceps muscle, anterior quadriceps muscle, and *M. pectoralis major* were 1.00 (CV = 0.82%), 0.99 (CV = 0.55%), and 0.99 (CV = 1.17%), respectively.

#### 2.6.1. Maximum dynamic strength test

Free weight 1-RM, SQ, and BP were used to determine the maximum dynamic strength at each test session (T0, T1, T2, T3) in the following order: 1) 1-RM SQ, 2) 1-RM BP. A power rack (Gym 80 International GmbH, Gelsenkirchen, Germany) with a 20 kg Olympic barbell (Gym 80 International GmbH, Gelsenkirchen, Germany) and competition plates (Gym 80 International GmbH, Gelsenkirchen, Germany) was used for the 1-RM SQ and BP assessments. Prior to testing, participants performed a standardized warm-up consisting of five minutes of low-intensity running, followed by a specific warm-up for free weight SQ. After the warm-up, participants were instructed to perform warm-up sets of 10, 5, 3 and 1 repetitions using 50%, 60%, 70% and 80% of the subjects’ estimated 1-RM. Between the warm-up sets, participants rest for 1, 2, 3 and 4 minutes, respectively. A weight close to the participant’s 1-RM was then used and, if the lift was successful, the weight was increased until the subject failed the attempt. A rest period of 4 minutes was maintained between 1-RM attempts. Once the 1-RM SQ was achieved, a 10 minute rest was taken and the same procedure was used to determine the 1-RM BP. For the SQ, a 1-RM attempt was considered successful if the participant started squatting from a standing position with the knees fully extended, lowered the bar to a position where the top of the hip was below the top of the knee joint, and the participant then fully extended the knees without any corrective movements. The BP 1-RM was considered successful if the participant maintained contact with the upper back, buttocks and both feet, touched the bar to the chest (no pause) and then fully extended the arms. Strong verbal encouragement from the researcher was given during all 1-RM attempts. The protocol chosen for 1-RM assessment was in accordance with the NSCA guidelines for 1-RM determination [59]. The ICC(3,1) and the CV for this population were 0.97 and 5.1% for the SQ, and 0.99 and 2.0% for the BP (unpublished data). ICC(3,1) was chosen as it represents test-retest reliability using a two-way mixed-effects model with absolute agreement, based on a single measurement per test session [60].

### 2.7. Resistance training protocol

The 12-week resistance training (RT) program for the EcDi and Plac groups consisted of three training sessions per week and followed a two-split training plan with three mesocycles of four weeks each. The two RT protocols (A/B) were trained alternately, with ABA in week one, BAB in week two, and so on. Each training day was followed by a rest day. Each mesocycle consisted of three weeks of loading phase and one deload week. The deload weeks were introduced to ensure adequate recovery for the 1-RM assessments and to counteract possible overloading due to rapid increases in training weights. Both training sessions consisted of six barbell, dumbbell or bodyweight exercises. Tables 1 and 2 show the protocols of the RT program with a detailed description of exercises, sets, repetitions, intensity (percentage (%) 1-RM; rate of perceived exertion (RPE)), tempo and rest.

**Table 1.** Resistance training protocol A.

Exercise	Cycle 1		Cycle 2		Cycle 3	
	Week 1–3 Loading	Week 4 Deload	Week 5–7 Loading	Week 8 Deload	Week 9–11 Loading	Week 12 Deload
	(sets ×repetitions)					
Back Squat	65% 1-RM	65% 1-RM	75% 1-RM	75% 1-RM	85% 1-RM	85% 1-RM
	3 ×12	1–2 ×12	3 ×8	1–2 ×8	3 ×5	1–2 ×5
Good Mornings	RPE 8–10	RPE 7	RPE 8–10	RPE 7	RPE 8–10	RPE 7
	3 ×15	2 ×15	3 ×12	2 ×12	3 ×10	2 ×10
Bench Press	65% 1-RM	65% 1-RM	75% 1-RM	75% 1-RM	85% 1-RM	85% 1-RM
	3 ×12	1–2 ×12	3 ×8	1–2 ×8	3 ×5	1–2 ×5
Dumbbell Flys	RPE 8–10	RPE 7	RPE 8–10	RPE 7	RPE 8–10	RPE 7
	3 ×15	2 ×15	3 ×12	2 ×12	3 ×10	2 ×10
Barbell Row	RPE 8–10	RPE 7	RPE 8–10	RPE 7	RPE 8–10	RPE 7
	3 ×15	2 ×15	3 ×12	2 ×12	3 ×10	2 ×10
Landmine Rotation	RPE 8–10	RPE 7	RPE 8–10	RPE 7	RPE 8–10	RPE 7
	3 ×15	2 ×15	3 ×12	2 ×12	3 ×10	2 ×10
Tempo (s)	2:0:1 (eccentric : isometric : concentric)					
Rest (s)	120–180 s (SQ, BP) and 90–120 s (all other lifts)					

1-RM, one repetition maximum; RPE, rate of perceived exertion; s, seconds; SQ, Squat; BP, Bench press;

**Table 2.** Resistance training protocol B.

Exercise	Cycle 1		Cycle 2		Cycle 3	
	Week 1–3 Loading	Week 4 Deload	Week 5–7 Loading	Week 8 Deload	Week 9–11 Loading	Week 12 Deload
	(sets ×repetitions)					
Dumbbell Lunges	RPE 8–10	RPE 7	RPE 8–10	RPE 7	RPE 8–10	RPE 7
	3 ×15	2 ×15	3 ×12	2 ×12	3 ×10	2 ×10
Romanian Deadlift	RPE 8–10	RPE 7	RPE 8–10	RPE 7	RPE 8–10	RPE 7
	3 ×12	1–2 ×12	3 ×8	1–2 ×8	3 ×5	1–2 ×5
Pull-ups	RPE 8–10	RPE 7	RPE 8–10	RPE 7	RPE 8–10	RPE 7
	3 ×12	1–2 ×12	3 ×8	1–2 ×8	3 ×5	1–2 ×5
Dumbbell incline Bench press	RPE 8–10	RPE 7	RPE 8–10	RPE 7	RPE 8–10	RPE 7
	3 ×15	2 ×15	3 ×12	2 ×12	3 ×10	2 ×10
Dumbbell Row	RPE 8–10	RPE 7	RPE 8–10	RPE 7	RPE 8–10	RPE 7
	3 ×15	2 ×15	3 ×12	2 ×12	3 ×10	2 ×10
Dumbbell Lateral Flexion	RPE 8–10	RPE 7	RPE 8–10	RPE 7	RPE 8–10	RPE 7
	3 ×15	2 ×15	3 ×12	2 ×12	3 ×10	2 ×10
Tempo (s)	2:0:1 (eccentric : isometric : concentric)					
Rest (s)	120–180 s (RDL, PU) and 90–120 s (all other lifts)					

RPE, rate of perceived exertion; s, seconds; RDL, Romanian Deadlift; PU, Pull-ups;

The weights for SQ and BP for each participant started at 65% of their achieved 1-RM at T0 during weeks 1–4 and were increased by 10% every 4 weeks. The weight of the % 1-RM based exercises was adjusted after each 1-RM test session. As a counterpart to the SQ and BP exercises in RT protocol A, the Romanian deadlift (RDL) and pull-ups (PU) were trained at the same intensity using the RPE scale and repetition scheme in RT protocol B over the 12-week period. With the exception of the SQ and BP, the load for all other exercises was determined by trial and error and standardized using the RPE scale to ensure appropriate intensity [61,62]. During the loading weeks, the final set of each exercise was performed until momentary concentric failure or until proper exercise technique could no longer be maintained.

The participants increased the training weights for lower body exercises every third training session (TS), based on the final set of the specific exercise using the following formula:

surplus repetitions TS1+surplus repetitions TS2=weight increase in kg

For upper body exercises, the following formula was used:

$$\frac{\left(\mathrm{surplus}\;\mathrm{repetitions}\;\mathrm{TS}1+\mathrm{surplus}\;\mathrm{repetitions}\;\mathrm{TS}2\right)}{2}=\mathrm{weight}\;\mathrm{increase}\;\mathrm{in}\;\mathrm{kg}$$

Proper exercise technique was always prioritized, and if this could not be ensured, the weight was not increased. All exercises were performed using the maximum range of motion achievable by each individual participant. The RT sessions were partially supervised by a certified member of the research staff. There was a 48 h break between the performance test and the RT program at T1 and T2 and a 72 h break before T0 and T3. The described RT program was previously used by Isenmann et al. [9], showing significant improvements in 1-RM for SQ and BP, as well as gains in muscle mass, with only minor adjustments made to the RT program for the current intervention.

### 2.8. Nutrition

Throughout the 12-week intervention, participants recorded their total dietary intake in a food diary using the FDDB app (Food Database GmbH, Bremen, Germany), which has been validated as a dietary assessment method [63]. After the baseline assessment, the subjects’ energy requirements were calculated using the Cunningham formula for resting energy expenditure (REE), and the physical activity level (PAL) was applied to determine total energy expenditure (TEE) [64]. Participants were instructed to eat in energy balance, and their TEE was provided to the subjects at the beginning of the intervention. Furthermore, subjects were required to consume 1.6–2.0 g of protein per kilogram of BW to ensure adequate recovery from the RT program [65].

#### 2.8.1. Supplements and dosage

In this study, a commercially available supplement (Weider Beta-Ecdysterone, LOD: 903176) was administered according to manufacturer’s recommendations (3 capsules per day). In previous research, the product analyzed for 20E content had a labeled concentration of 25 mg per capsule, with 22.29 mg (89.16% of the labeled amount) actually detected [29]. Therefore, this supplement demonstrated the highest total concentration and content of 20E per capsule [29]. According to the manufacturer’s label, the product (Weider Beta-Ecdysterone) contains 25 mg 20E from spinach extract, 50 mg DSG from yam root extract, 250 mg γ-oryzanol, 0.07 mg vitamin B6, 0.03 mg folic acid and 0.3 mg pantothenic acid per capsule. In addition, the new batch of the product was analyzed for its 20E content after the study. For validation, capsules from the same batch used by Ambrosio et al. [29] were also analyzed. The placebo consisted of a gelatin capsule and 1 g of sugar and was prepared by a member of the research team. Each individual in the Plac group took three capsules of the placebo compound per day. In both groups, the capsules were taken as follows: one in the morning, one at noon, and one in the evening. All participants in both groups were not allowed to talk about the supplement or open up the capsules to ensure blinding. The first dose consisted of 28 daily servings and was given to the subjects on the first day of the intervention. The next two doses (28 daily servings) were given to the participants after T1 and T2.

### 2.9. Quantitative supplement analysis

Quantitative analysis was performed by UHPLC-MS/MS on an Agilent 1290 Infinity II UHPLC coupled to an Agilent 6495A triple quadrupole (QQQ) tandem MS system for the analysis of 20E or to an Agilent 6550A quadrupole time of flight (QToF) tandem MS system (Agilent Technologies GmbH, Waldronn, Germany) for the analysis of DSG. MassHunter software (Agilent Technologies GmbH, Waldronn, Germany) was used for data acquisition and quantitative analysis.

Chromatographic separation was achieved on an Agilent Poroshell EC-C18 column (50×2.1 mm; 1.9 µm particle size) with solvent A consisting of H_2_O:formic acid (99.9:0.1, v:v) and solvent B of ACN:formic acid (99.9:0.1, v:v). While for DSG, an isocratic elution for 5 min at 20 % A/80 % B [https://doi.org/10.1155/2022/5607347] was employed, a stepwise linear gradient was used for 20E analysis: 5 % B for 1 min, 0.2 min increase to 19 % B, 2.8 min increase to 35 % B, 0.2 min increase to 98 %, 2 min hold and 0.3 min re-equilibration to 5 % B followed by 2 min of post run time. Sample injection volume was set to 2 µL (20E)/5 µL (DSG), flow rate to 0.5 mL/min and column temperature to 30 °C (20E)/35 °C (DSG).

Both MS systems utilized a jet stream electrospray ionization source operated in positive mode (ESI+). Ion source parameters have previously been reported by Ambrosio et al. [29]. For DSG analysis capillary voltage and nozzle voltage were adapted to 3000 V and 2000 V, respectively. The QQQ was operated in multiple reaction monitoring (MRM) mode with the transitions reported in Table 3, whereas the QToF ran in MS1 scan mode. For quantitation of DSG, [M+H]^+^ was used with the extraction window set to ±10 ppm.

**Table 3.** MRM transitions.

Substance	Fragment	Precursor Ion (m/z)	Product Ion (m/z)	Collision Energy	Cell Accelerator Voltage
Ecdysterone	Quantifier	481	445	12	5
	Qualifier 1		371	12	4
	Qualifier 2		165	28	5
Ponasterone (internal standard)	Quantifier	465	80.9	44	3
	Qualifier 1		173	24	6
	Qualifier 2		109	32	6

The supplement batch used in this trial (L:903176) was tested as well as the previous batch (L: 402305) already analyzed by Ambrosio et al. [29]. Six capsules of each batch were opened, their content weighted and mixed. Three times 100 mg of capsule content were dissolved in 10.0 mL DMSO:EtOH:H_2_O (50:30:20, v:v:v) each and subsequent extraction was carried out analogously to Ambrosio et al. [29].

To account for the unknown matrix effect, quantitation was performed by standard addition. The extracts were diluted with LC-MS grade methanol. Aliquots of 10 µL 20E standard solution (0; 10; 20; 30; 40; 50; 60 ng/mL) and 10 µL ponasterone standard solution (100 ng/mL) as internal standard were added to 80 µL of diluted extract (1:10 dilution). For DSG 10 µL of standard solution (0; 500; 1000; 1500; 2000; 2500 ng/mL) were added to 90 µL of diluted extract (1:10000 dilution). All calibration points were prepared in triplicate.

#### 2.9.1. Myotube measurement of C2C12 cell

Following the training intervention, the commercially available supplement used in this study (Ecdy & Dio Mix 2024) was analyzed for its biological activity and potential anabolic effects in a well-established C2C12 cell line-based assay, that has been used in numerous other studies [7,21,66–68]. C2C12 cells, a myoblast cell line derived from murine satellite cells, were used as an *in vitro* model to study muscle fiber hypertrophy induced by this supplement. The effects of this supplement on myotube diameter were compared with the control, dihydrotestosterone (DHT), as well as the same batch of the supplement (Ecdy & Dio Mix. 2018) previously analyzed by Ambrosio et al. [29].

#### 2.9.2. Statistical analysis

All analyses were performed using R statistical software version 4.4.1 and R Studio [69]. Results are presented as mean ± standard deviation (SD) unless otherwise stated. Baseline characteristics of the participants and nutritional data from the food diary were tested for normal distribution using the Shapiro-Wilk test. If normal distribution was confirmed, a Welch two-sample t-test was used for further analyses. In cases where the data did not confirm normal distribution (body weight at baseline testing T0), the Wilcoxon test was used. The blood sample, body composition, muscle thickness, and 1-RM data were tested and checked for normal distribution using both residuals vs. fitted plots and Q-Q plots. All dependent variables from the blood samples, body composition, muscle thickness and 1-RM tests were analyzed using linear mixed model (LMM) with the lme4 and the lmerTest packages [70,71]. For all LMM, the fixed effects of group (EcDi, Plac), time (T0, T1, T2, T3), and their respective interaction terms are incorporated into each model. In the LMM models for blood samples and muscle thickness assessments, only T0 and T3 were used for the fixed effects of time. The emmeans package [72] was used to fit the models. Post hoc tests were then calculated for the appropriate model with false discovery rate (FDR) adjustment, and a pairwise comparison of the changes (Δ) in both groups was performed for each variable. The significance threshold for all analyses was set at a p-value < 0.05. Effect sizes were estimated using Cohen’s *d* and classifications were categorized as follows: trivial < 0.2; small < 0.5; moderate < 0.8; strong > 0.8 [73]. Figures were generated using R statistical software with the ggplot2 package [74].

## 3. Results

After dropouts, 24 participants (EcDi, *n* = 12; Plac, *n* = 12) completed the study and were included in the analyses. Reasons for dropout are shown in the participants flowchart (Figure 1). The baseline characteristics of the subjects can be seen in Table 4. There were no significant differences between the groups at baseline.

**Table 4.** Baseline characteristics.

	EcDi (*n* = 12)	Plac (*n* = 12)	p-values
Age (y)	31.7 ± 7.2	30.5 ± 9.3	.733
Height (cm)	183.0 ± 7.0	180.7 ± 9.5	.501
Bodyweight (kg)	90.4 ± 13.1	88.5 ± 15.1	.686
Training Age (y)	8.5 ± 5.1	10.4 ± 5.6	.380
Relative strength (SQ)	1.43 ± 0.23	1.50 ± 0.30	.538
Relative strength (BP)	1.21 ± 0.22	1.21 ± 0.23	.972

Data are shown as mean ± standard deviation; SQ, Squat; BP, Bench press; y, years; p-values from Welch two Sample t-test and in case of BW Wilcoxon test.

### 3.1. 1-RM tests

Results of the 1-RM tests over the 12 weeks are reported in Table 5.

**Table 5.** 1-RM SQ and BP T0-T3.

Metric	Group	T0	T1	T2	T3	p-values time	p-values group
1-RM SQ (kg)	EcDI	128.3 ± 22.4	132.9 ± 23.5	136.3 ± 22.6	141.0 ± 20.9	< 0.05*	n.s.
	Plac	130.8 ± 24.3	136.0 ± 22.6	138.9 ± 19.8	143.8 ± 18.8		
1-RM BP (kg)	EcDI	107.7 ± 18.5	107.3 ± 18.8	108.8 ± 19.1	110.8 ± 18.1	< 0.05*	n.s.
	Plac	105.0 ± 15.2	105.8 ± 15.7	105.9 ± 16.1	107.3 ± 16.4		
RS SQ	EcDI	1.43 ± 0.23	1.49 ± 0.24	1.52 ± 0.22	1.58 ± 0.23	< 0.05*	n.s.
	Plac	1.50 ± 0.30	1.56 ± 0.28	1.59 ± 0.23	1.63 ± 0.28		
RS BP	EcDI	1.21 ± 0.22	1.21 ± 0.22	1.22 ± 0.22	1.24 ± 0.22	< 0.05*	n.s.
	Plac	1.21 ± 0.23	1.22 ± 0.24	1.24 ± 0.24	1.25 ± 0.25		

Data are shown as mean ± standard deviation; SQ, Squat; BP, Bench press; n.s., not significant; *Significance level at *p* ≤ 0.05.

**Figure 3.**
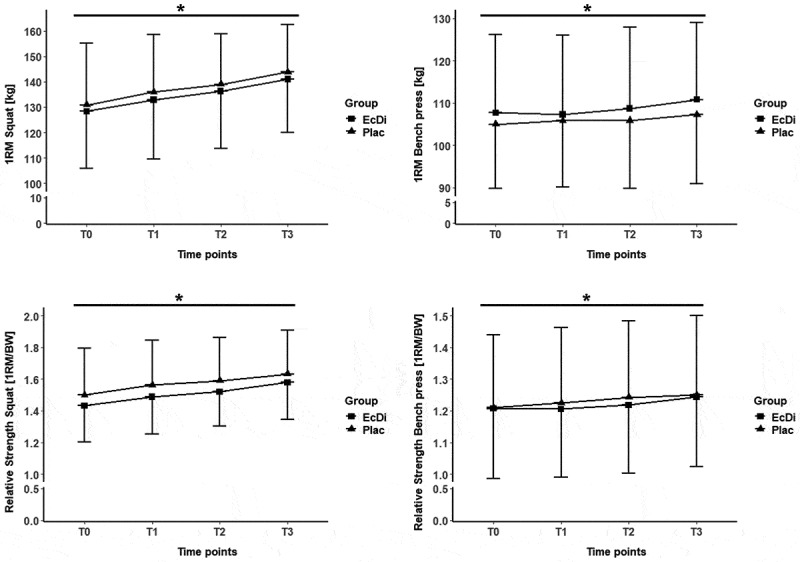
1-RM SQ and BP T0-T3. *significance level at *p* ≤ 0.05; time effects were marked with *.

Figure 3 illustrates the development of the 1-RM SQ and BP strength, along with the relative strength progression for both exercises.

At baseline (T0), no significant differences were found between the groups for 1-RM SQ and BP (all *p* > 0.05). The LMM analysis revealed a significant time effect for both 1-RM SQ and BP. For SQ, a significant time effect (all *p* < 0.05) was observed at T1, T2, and T3. However, for BP, a significant effect of time was only found at T3. In addition, no significant group effect or time × interactions were detected.

The estimation of Cohen’s *d* indicated a moderate effect size (*d* = 0.60) for the significant increase in 1-RM SQ and a trivial effect size (*d* = 0.16) for 1-RM BP from T0 to T3.

The LMM showed no significant difference between both groups for relative strength in SQ and BP (all *p* > 0.05). Similar to the significant time effect observed for 1-RM SQ and BP, a significant time effect was found for relative strength in SQ at T1, T2, and T3, and for BP only at T3. No significant group effects or time × interactions were detected. Analysis of effect sizes revealed a moderate effect for relative strength in SQ (*d =* 0.55) and a small effect for relative strength in BP (*d* = 0.16) in the increase from T0 to T3.

#### 3.1.1. Body composition

The LMM showed no significant differences between groups at T0 for BW (*p* = 0.74), FFM (*p* = 0.32), FM (*p* = 0.50), MM (*p* = 0.58), TBW (*p* = 0.31), and BF% (*p* = 0.22). The development of the body composition variables from T0 to T3 is shown in Table 6.

**Table 6.** Body composition variables T0-T3.

Metric	Group	T0	T1	T2	T3	p-values time	p-value group
Body-weight (kg)	EcDi	90.4 ± 13.1	89.9 ± 12.6	90.2 ± 12.8	90.1 ± 12.7	.142	n.s.
	Plac	88.5 ± 15.1	88.3 ± 14.3	88.5 ± 14.6	89.7 ± 15.5		
Fat free mass (kg)	EcDi	71.4 ± 8.3	72.1 ± 8.4	72.5 ± 8.0	72.0 ± 7.9	.003*	n.s.
	Plac	67.7 ± 9.4	69.3 ± 9.2	68.8 ± 9.9	69.5 ± 10.4		
Fat mass (kg)	EcDi	18.9 ± 6.3	17.9 ± 5.9	17.7 ± 6.4	18.1 ± 6.3	.048*	n.s.
	Plac	20.9 ± 7.7	19.1 ± 6.9	19.8 ± 6.9	20.2 ± 7.3		
Muscle mass (kg)	EcDi	49.3 ± 5.9	49.9 ± 5.7	50.4 ± 5.0	50.2 ± 5.5	.002*	n.s.
	Plac	47.9 ± 6.9	48.7 ± 6.7	49.1 ± 7.3	49.0 ± 7.6		
Total body water (L)	EcDi	52.3 ± 6.1	52.8 ± 6.2	53.0 ± 5.9	52.3 ± 5.3	.025*	n.s.
	Plac	49.5 ± 6.9	50.7 ± 6.7	50.3 ± 7.3	50.9 ± 7.6		
Body fat p. (%)	EcDi	20.6 ± 4.4	19.5 ± 4.3	19.2 ± 4.9	19.7 ± 4.5	.029*	n.s.
	Plac	23.1 ± 5.5	21.2 ± 4.7	21.9 ± 5.1	22.2 ± 5.1		

Data are shown as mean ± standard deviation; n.s., not significant; *Significance level at p ≤ 0.05.

**Figure 4.**
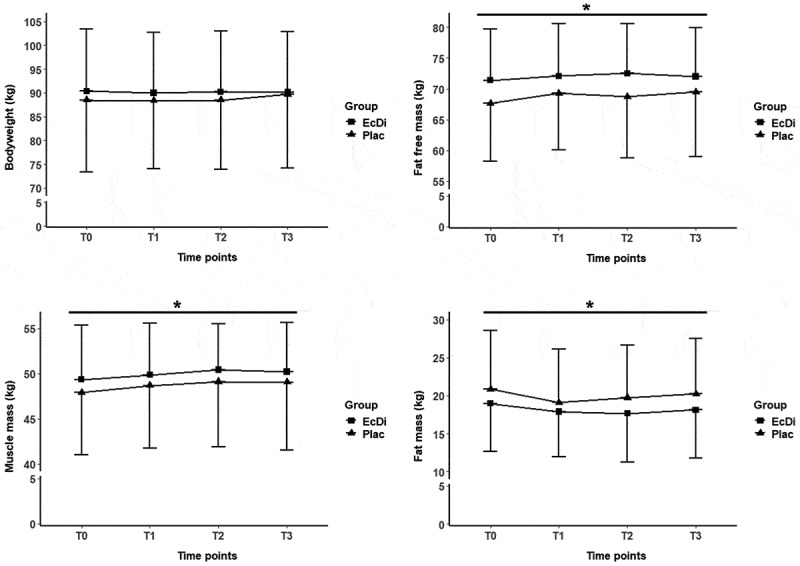
Bodyweight, fat free mass, muscle mass, fat mass, total body water and body fat percentage T0-T3. *significance level at *p* ≤ 0.05; time effects were marked with *.

**Figure 5.**
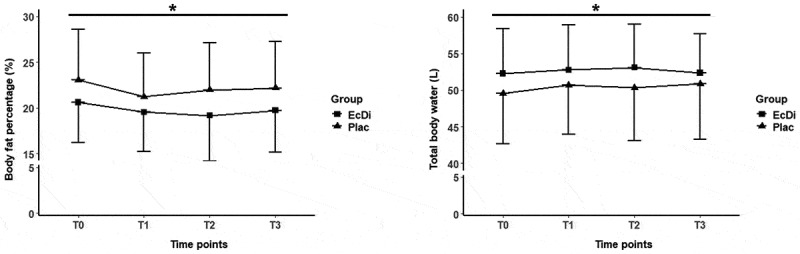
Total body water and body fat percentage T0-T3. *significance level at *p* ≤ 0.05; time effects were marked with *.

Figures 4 and 5 illustrate the changes in the body composition variables from T0 to T3.

Figure 4 and 5 are only one Figure (4)

Figure 4 and 5 should be summarized to figure 4

For BW, the LMM showed no significant main effect of time (*p* > 0.05). FFM showed significant increases over time, with values at T1, T2, and T3 higher than at baseline (T0) (all *p* < 0.05). However, no significant differences were found between T1, T2, and T3 (all *p* > 0.05), indicating that FFM levels plateaued after the initial increase. FM decreased significantly over time, with reductions observed at T1 and T2 (both *p* < 0.05) and a trend toward significance at T3 (*p* = 0.09). No significant differences were found between T1, T2, and T3 (all *p* > 0.05), suggesting that the decrease occurred primarily between T0 and T1. BF% also decreased significantly over time, with reductions at T1 and T2 (both *p* < 0.05) and a trend at T3 (*p* = 0.09). No significant differences were found between T1, T2, and T3 (all *p* > 0.05), suggesting that the decrease in BF% occurred mainly in the early stages, similar to FM. MM increased significantly over the study period at all time points (all *p* < 0.05). TBW also increased significantly at all time points (all *p* < 0.05). While the LMM showed significant effects for FFM, FM, BF%, MM and TBW over time, no group effects or time × group interactions were observed for any of the variables (all *p* > 0.05).

Calculations of Cohen’s *d* for FFM (d = 0.13), FM (d = 0.10), MM (d = 0.15), BF% (d = 0.18), and TBW (d = 0.10) indicated trivial effect sizes for the main effect of time.

#### 3.1.2. Muscle thickness

**Figure 6.**
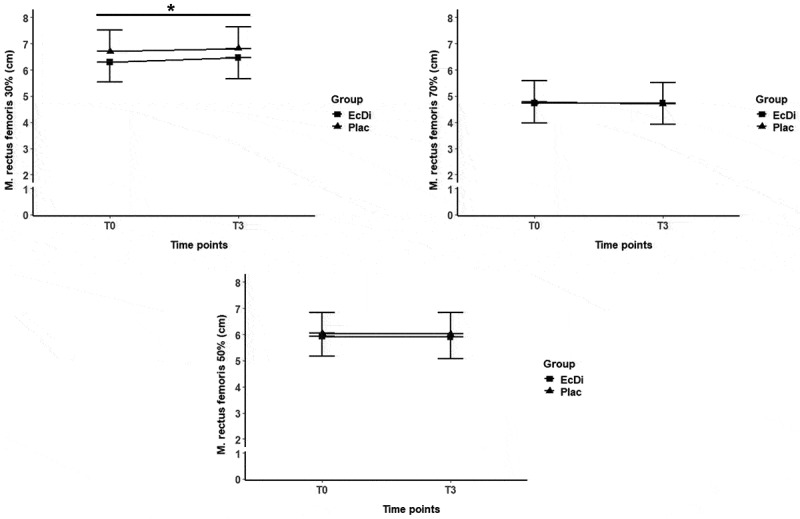
Anterior quadriceps femoris muscle thicknessT0-T3. *significance level at *p* ≤ 0.05; time effects were marked with *.

**Figure 7.**
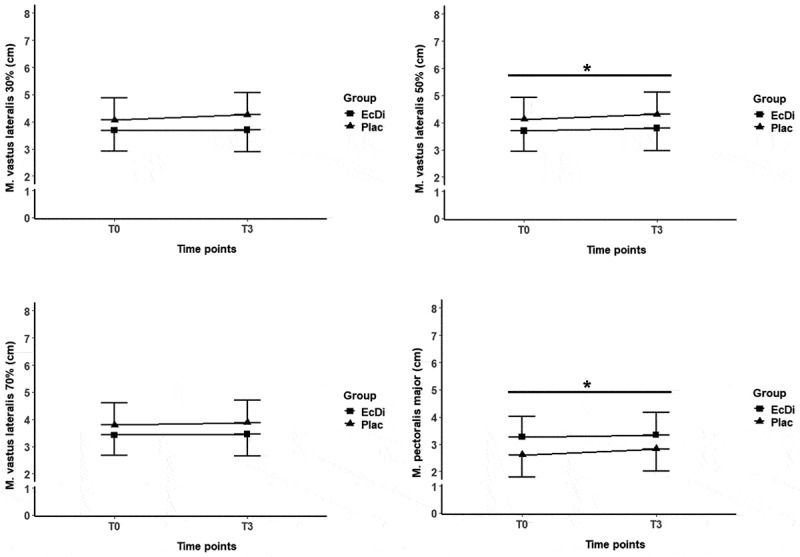
Muscle thickness of lateral quadriceps femoris and M. pectoralis major T0-T3. *significance level at *p* ≤ 0.05; time effects were marked with *.

Table 7 presents the development of muscle thickness for RF, VL, and PM from T0 to T3, with changes illustrated in Figures 6 and 7.

**Table 7.** Muscle thickness T0-T3.

Parameter	Group	T0	T3	P-values time	p-values group
Anterior quadriceps femoris 30% (cm)	EcDi	6.3 ± 0.8	6.6 ± 0.8	.011*	n.s.
	Plac	6.7 ± 0.8	6.8 ± 0.8		
Anterior quadriceps femoris 50% (cm)	EcDi	5.9 ± 0.9	5.9 ± 0.7	.783	n.s.
	Plac	6.0 ± 0.9	6.0 ± 0.8		
Anterior quadriceps femoris 70% (cm)	EcDi	4.7 ± 0.7	4.7 ± 0.7	.501	n.s.
	Plac	4.8 ± 0.9	4.7 ± 0.8		
Lateral quadriceps femoris 30% (cm)	EcDi	3.7 ± 0.9	3.7 ± 0.7	.086	n.s.
	Plac	4.1 ± 1.1	4.3 ± 1.1		
Lateral quadriceps femoris 50% (cm)	EcDi	3.7 ± 0.7	3.8 ± 0.5	.020*	n.s.
	Plac	4.1 ± 1.0	4.3 ± 0.8		
Lateral quadriceps femoris 70% (cm)	EcDi	3.4 ± 0.5	3.5 ± 0.4	.118	n.s.
	Plac	3.8 ± 0.8	3.9 ± 0.6		
M. pectoralis major (cm)	EcDi	3.3 ± 0.6	3.3 ± 0.7	.008*	n.s.
	Plac	2.6 ± 0.5	2.8 ± 0.6		

Data are shown as mean ± standard deviation; n.s., not significant; *Significance level at p ≤ 0.05.

##### 3.1.2.1. Anterior quadriceps femoris muscle thickness

At baseline (T0), no significant differences (*p* > 0.05) were observed between both groups for anterior quadriceps femoris muscle thickness at the 30%, 50% or 70% landmarks. The LMM model showed a significant increase (*p* < 0.05) in anterior quadriceps femoris muscle thickness at 30% over time. However, no group effect or time × group interaction was observed (all *p* > 0.05). The calculation of Cohen’s d for anterior quadriceps femoris thickness at 30% was 0.17, indicating a trivial effect size.

##### 3.1.2.2. Lateral quadriceps femoris muscle thickness

Similarly, no significant differences (all *p* > 0.05) were found between the EcDi and Plac groups at baseline (T0) for the lateral quadriceps femoris muscle thickness at the 30%, 50%, or 70% landmarks. A significant time effect was observed for lateral quadriceps femoris at 50% landmark (*p* < 0.05), whereas no significant changes were found at the 30% or 70% landmarks. Additionally, no significant group effect or time × group interaction (all *p* > 0.05) was detected. The calculation of Cohen’s d for lateral quadriceps femoris thickness at 50% was 0.17, indicating a trivial effect size.

##### 3.1.2.3. M. pectoralis major muscle thickness

In contrast, at baseline (T0), a significant group difference (*p* < 0.05) was observed between the EcDi and Plac groups for M. pectoralis major. The LMM analysis revealed a significant time effect (*p* < 0.05) for PM muscle thickness; however, no significant group effect or time × group interaction was found. Cohen’s *d* for the change in PM thickness between T0 and T3 was 0.38, indicating a small effect size.

#### 3.1.3. Liver and kidney biomarkers

After analysis of liver and kidney biomarkers, creatinine, GOT, and GGT showed no significant main effects of group or time, nor any significant group × time interaction effects (*p* > 0.05). For GPT, the LME model revealed a significant main effect of group, with the EcDi group having lower GPT levels compared to the Plac group at T0 (*p* < 0.05). There was also a trend toward a significant effect of time (*p* = 0.09), suggesting a decrease in GPT levels over time in the Plac group. Furthermore, an initial significant group × time interaction was observed (*p* < 0.05), but this effect was no longer significant after FDR adjustment.

#### 3.1.4. Hormone concentrations

Throughout the 12-week intervention period the hormone concentrations of E2, T, fT, CORT, and IGF1 showed no significant main effects for group or time, nor any significant group × time interaction effects (*p* > 0.05). The raw data for liver and kidney function and the various hormone parameters are shown in the Supplementary Appendix.

#### 3.1.5. Nutritional behaviour

After the 12-week intervention period, analysis of the food diaries using the Welch two-sample t-test showed no significant differences in any of the provided macronutrients or daily energy intake. Table 8 shows the mean values for macronutrients and kilocalories, as well as the relative protein intake per kilogram of BW over the 12-week study period.

**Table 8.** Nutrition of the participants.

Macronutrient	EcDi	Plac	P-values
Energy intake (kcal/day)	2697 ± 299	2522 ± 511	.404
Fat (g/day)	89 ± 21	82 ± 26	.580
Carbohydrates (g/day)	283 ± 43	267 ± 73	.584
Protein (g/day)	174 ± 24	162 ± 26	.317
Protein (g/kg BW)	1.9 ± 0.4	1.8 ± 0.3	.306

Data are shown as mean ± standard deviation; BW p-values from the Welch Two Sample t-test.

#### 3.1.6. Diameter of C2C12 cells

**Figure 8.**
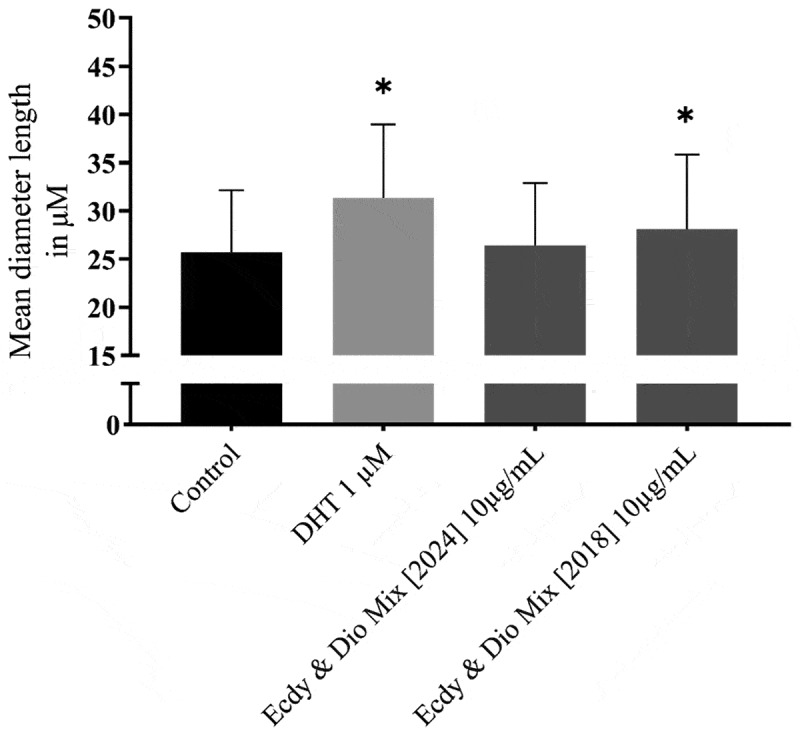
Results for the C2C12 cell line; *significance level at *p* < 0.05; treatment effects were marked with*.

According to the analysis of C2C12 cells, the commercially available supplement (Ecdy & Dio Mix 2024) revealed no significant hypertrophic effects on myotube diameter. Table 9 shows the myotube diameter after treatment with the different batches of the supplement. As shown in Figure 8, both DHT and the earlier batch of the supplement (Ecdy & Dio Mix 2018) showed a significant increase in diameter length compared to the control.

**Table 9.** Myotube diameter after treatment with the supplements.

Substances	$\overset{ˉ}{x}$ in µM	% of Control	P value
Control	25,7 ± 6,4	100	
Dihydrotestosterone (DHT)	31,3 ± 7,6	122	< .05
Ecdy & Dio Mix [2018]	28,1 ± 7,7	110	< .05
Ecdy & Dio Mix [2024]	26,4 ± 6,5	103	n.s.

Data are shown as mean ± standard deviation. n.s. not significant.

### 3.2. Supplement analysis

For the new batch, less than 10 µg 20E and 15.6 mg DSG per capsule were detected, equivalent to less than 0.1% and 31.2% of the packaging’s claim. In contrast, analysis of the old batch confirmed the previous findings of Ambrosio et al. [26] for 20E and revealed a content of 40.4 mg DSG per capsule, accounting for 80.8% of packaging’s claim.

## 4. Discussion

This study aimed to investigate the potential anabolic and performance-enhancing effects of a commercially available phytosteroid product labeled to contain 20E and DSG. The training protocol was shown to increase upper and lower body strength as well as FFM and MM. Analyses of the commercially available product revealed that the actual 20E content was less than 0.1% while the DSG content was only 10.4% of the amount claimed by the manufacturer. Consequently, no hypertrophic effects were observed in the C2C12 model, and no difference was detected between the two human intervention groups. However, the *in vitro* model showed that anabolic effects were achieved when 20E and DSG were included in the product, as demonstrated by the commercial product of 2018.

In terms of performance, the training protocol was shown to induce a significant increase in 1-RM SQ and BP performance. The increases in both exercises were consistent with a previous study using a similar training protocol [7]. A significant moderate effect was observed for 1-RM SQ and a significant trivial effect for 1-RM BP in both groups. Considering the performance level of the participants in this study, it can be classified as slightly higher than that of a previous study [7]. Therefore, the potential for adaptation in terms of performance, as well as an increase in FFM, MM and muscle thickness, was lower in the population examined in this study. Nevertheless, the results are similar to those of the previous investigation [7].

Consistent with these findings, the present study also observed significant changes in FFM, MM, and TBW, along with decreases in FM and BF%. Effect sizes for body composition were trivial, which is reasonable given the participants’ advanced training levels. Results align with the meta-analysis by Benito et al. [75], who reported mean increases of 1.6 kg in FFM and 1.1 kg in MM in response to RT programs, further supporting the findings of the present study. Furthermore, analysis of the muscle thickness data showed a significant main effect of time on the anterior quadriceps muscle thickness at 30% and lateral quadriceps muscle thickness at 50%, both of which had a trivial effect size (d = 0.17). When compared with similar muscle thickness measurements by Gavanda et al. [54] over a 12-week period, the absolute increases in *M. rectus femoris* (Block: 0.11 cm; DUP: 0.20 cm) and *M. vastus lateralis* (Block: 0.13 cm; DUP: 0.10 cm) thickness are similar. Taken together, these findings provide plausibility for the significant increases in FFM and MM and support the efficacy of the RT program, suggesting that it can be reliably used in future studies.

However post-study analysis of the supplement revealed a 20E content of less than 0.1% of the amount claimed by the manufacturer on the label (25 mg 20E). The content differs significantly from that of the old batch of the same product, but illustrates the wide variance in the actual 20E concentration in commercially available ecdysterone products as also reported by Ambrosio et al. [29]. These findings raise important considerations, as dietary supplements are widely used by both the general population and athletes. The Federal Office for Consumer Protection and Food Safety in Germany states that dietary supplements do not require proof of efficacy and that the manufacturer is responsible for safety [30]. In addition, the amount of ingredients listed on the label can vary by as much as 50% from the actual amount of ingredients [30]. Due to the lack of regulation, many dietary supplements on the market are not analyzed for the content of certain ingredients. Several studies of the accuracy of dietary supplement labeling have found that many of the products analyzed contained significantly lower amounts of the specific ingredient or additional, possibly risky, ingredients that were not declared on the supplement label [29,32,33,76–81]. Similar to Germany, dietary supplements in the U.S. do not require proof of efficacy and are assumed to be safe, unless proven otherwise [82]. According to Navarro et al. [83], herbal and dietary supplements (HDS) accounted for 20% of liver injuries in 2013–2014 due to contamination with anabolic steroids or toxic potential of other ingredients. Contamination of dietary supplements is not only a health problem for the general population. Kozhuharov et al. [84] conducted a systematic review and showed that more than 28% of the analyzed dietary supplements posed a potential risk of unintentional doping. This is supported by previous investigations [33]. For professional athletes in particular, this may be career-terminating due to a suspension by the WADA and public standing. Irrespective of the potential risks of side effects due to inaccurate amounts of ingredients and potential impurities, the sometimes contradictory observations of the potency of 20E in humans may be explained.

Initial studies by Wilborn et al. failed to demonstrate any additive effects of chronic application of 100 mg Polypodium Vulare/Suma root on performance and body composition after 8 weeks of strength training [25]. According to the information provided at the time, the capsules should contain 30 mg 20E. However, no information was provided on the analyses of the supplements used and the actual concentration [25]. Studies on foods such as spinach or quinoa, which generally contain 20E, show that the concentration is highly dependent on various factors such as origin, genetics and environmental conditions [38,85–90]. Similar to this study, the extract used in the investigations by Wilborn et al. at that time may not have contained any significant biologically active concentrations of 20E [25]. In contrast, *in vitro* studies and animal models with the pure substance 20E show clear anabolic effects [16–21]. In addition, anabolic effects, improvements in muscle quality and performance-enhancing effects have been observed in human studies [7,24]. Even though the actual concentration of the product used in the study by Isenmann et al. also deviated significantly from the specified concentration, anabolic effects were demonstrated in the cell culture [7]. Furthermore, the actual concentrations used per week (low dose: 72 mg/week, high dose: 336 mg/week) by Isenmann et al. were within the range of classic anabolic substances [91]. Besides, comprehensive screening for prohibited substances was conducted on both the administered product and the participants, in compliance with current WADA guidelines. In contrast, the weekly dosage in the study by Pérez-Piñero et al was considerably lower (22.4 mg/week) than that reported by Isenmann et al. While some anabolic steroids exert anabolic effects at doses as low as approximately 10 mg per week, Pérez-Piñero et al. suggest that the high nitrate concentration (14.18 mg/day, 99,26 mg/week) may be primarily responsible for the observed biological activity [24]. Consequently, the anabolic effects cannot be clearly attributed to the 20E concentration. Although spinach (~900 g) and quinoa (150 g) contained approximately 18–19 mg and 55.3 mg of 20E, respectively, only a small proportion (1–3%) was absorbed, rendering significant biological activity unlikely [39,41]. While anabolic effects have been unequivocally demonstrated *in vitro* and in animal models [14,16,18–21], and partially observed in humans [7], current data remain insufficient to define precise dose – response relationships.

Unlike the analysis of 20E, no method has yet been established for determining DSG in supplements. The present study determined the DSG concentration in supplements for the first time. Similar to 20E, the concentration of DSG varied considerably among the tested products. In the product used for this investigation, only approximately 30% of the declared content was present in the capsules. Based on the very low concentrations of both phytosteroids, no anabolic effects were observed in either the cell culture or the human experiment. In previous cell culture experiments by Kostov et al., a dose-response relationship was identified for both phytosteroids [21]. Moreover, similar effects were also observed in humans. Studies in both men and women have observed higher effects on MM with higher doses of DSG-rich fenugreek [47,92]. Comparing the DSG data of the product used here, however, they are lower than those reported in previous studies [47,92].

Interestingly, the results of 20E, DSG Mix from 2018 in the cell culture experiments were similar to the effects by Kostov et al. for the ratio of 1:2 (20E:DSG) [21]. It appears that a combination of 1:2 20E to DSG has no additive effect. Based on the findings of Kostov et al., two different mechanisms of action are activated, but additive effects are primarily observed at ratios of 2:1; 10:1 or 1:10 20E to DSG [21]. As a result, exact combination ratios have not yet been sufficiently investigated.

In summary, it is currently not possible to comment on the possible additive effects of 20E and DSG in humans due to the almost complete absence of 20E and DSG in the study supplement. Therefore, for future studies with phytosteroids in general, the following points need to be considered to identify potential effects in general:

1. Verification of the product content and absence of potential contamination with prohibited substances,
2. Verification of biological activity *in vitro* models,
3. Human intervention study with blood and urine analyses regarding bioavailability of the substances.

If these recommendations are followed in the studies, outcomes similar to those observed in this study are less likely to recur.

## 5. Limitations

The primary limitation of this study is that the supplement used was not analyzed prior to the start of the investigation to ensure that it contained a sufficiently high amount of 20E and DSG, which could potentially have performance-enhancing and anabolic properties. Although a previously tested product was used, relying on older analyses is insufficient; each batch must be independently tested to ensure consistency in substance concentration. As long as regulatory guidelines remain vague, these issues are likely to persist. Besides, this study included only a small sample size of male participants, so no conclusions can be drawn regarding female responses. Although body composition assessment using BIA is considered valid, the current gold standard remains dual-energy X-ray absorptiometry (DXA). However, this is scarcely feasible in Germany with healthy people.

## 6. Conclusion

Based on the significant time effects observed in 1-RM performance, body composition variables, and muscle thickness data, the RT program used in this study appears to be effective and can serve as a foundation for future research. However, given the minimum 20E and DSG content found in the supplement, there is a clear need for stringent guidelines for future phytosteroid studies. Sufficient testing of study materials prior to initiating human studies would reduce the likelihood of outcomes similar to those observed in this study. Furthermore, policymakers should establish more specific regulations to ensure that consumers are not misled by manufacturers’ claims and to prevent adverse health effects from incorrect labeling of ingredient concentrations.

## Data Availability

Anonymized raw data can be viewed and obtained upon request from the corresponding author.

## Abbreviations

The following abbreviations are used in this manuscript:

1-RM: One-Repetition Maximum
20E: 20-hydroxyecdysone/ecdysterone
BW: Body weight
BF%: Body fat percentage
CORT: Cortisol
CV: Coefficient of variation
DSG: Diosgenin
DXA: Dual-energy x-ray absorptiometry
EcDi: Ecdysterone and diosgenin group
E2: Estradiol
ERβ: Estrogen receptor β
FM: Fat mass
FFM: Fat-free mass
FDR: False discovery rate
fT: Free testosterone
GGT: Gamma-glutamyltransferase
GPCR: G-protein coupled receptor
GPT: Glutamate pyruvate transaminase
GOT: Glutamate oxaloacetate transaminase
ICC: Intraclass correlation coefficient
IGF1: Insuline like growth factor 1
LMM: Linear mixed model
MM: Muscle mass
PAL: Physical activity level
Plac: Placebo group
PM: M. pectoralis major
PU: Pull-ups
RDL: Romanian Deadlift
REE: Resting energy expenditure
RF: M. rectus femoris
RPE: Rate of perceived exertion
RT: Resistance training
T: Testosterone
TBW: Total body water
TEE: Total energy expenditure
TS: Training session
VL: M. vastus lateralis
WADA: World Anti Doping Agency

## References

[1] Knapik JJ, Steelman RA, Hoedebecke SS, et al. Prevalence of dietary supplement use by athletes: systematic review and meta-analysis. Sports Med. 2016;46(1):103–23. doi: 10.1007/s40279-015-0387-7 26442916 PMC4697915

[2] Isenmann E, Tolle P, Geisler S, et al. Differences in consumption behaviour of dietary supplements in competitive athletes depends on Sports discipline. Nutrients. 2024;16(3):374. doi: 10.3390/nu16030374 38337659 PMC10857381

[3] Sánchez-Oliver AJ, Domínguez R, López-Tapia P, et al. A survey on dietary supplement consumption in amateur and professional rugby players. Foods. 2020;10(1):7. doi: 10.3390/foods10010007 33375061 PMC7822035

[4] Moreno B, Veiga S, Sánchez-Oliver AJ, et al. Analysis of Sport supplement consumption by competitive swimmers according to sex and competitive level. Nutrients. 2022;14(15):3218. doi: 10.3390/nu14153218 35956394 PMC9370690

[5] Halabchi F, Shab-Bidar S, Selk-Ghaffari M. Prevalence of supplement consumption in Iranian athletes: a systematic review and meta-analysis. Int J Prev Med. 2021;12(1):32. doi: 10.4103/ijpvm.IJPVM_189_20 34249281 PMC8218796

[6] Garthe I, Maughan RJ. Athletes and supplements: prevalence and perspectives. Int J Sport Nutr Exerc Metab. 2018;28(2):126–138. doi: 10.1123/ijsnem.2017-0429 29580114

[7] Isenmann E, Ambrosio G, Joseph JF, et al. Ecdysteroids as non-conventional anabolic agent: performance enhancement by ecdysterone supplementation in humans. Arch Toxicol. 2019;93(7):1807–1816. doi: 10.1007/s00204-019-02490-x 31123801

[8] Isenmann E, Alisauskas P, Flenker U, et al. The anabolic effect of fenugreek: a systematic review with meta-analysis. Int J Sports Med. 2023;44(10):692–703. doi: 10.1055/a-2048-5925 37253363

[9] Albaker WI. Fenugreek and its effects on muscle performance: a systematic review. J Pers Med. 2023;13(3):427. doi: 10.3390/jpm13030427 36983608 PMC10054907

[10] Parr MK, Botrè F, Naß A, et al. Ecdysteroids: a novel class of anabolic agents? Biol Sport. 2015;32(2):169–173. doi: 10.5604/20831862.1144420 26060342 PMC4447764

[11] Dinan L, Dioh W, Veillet S, et al. 20-hydroxyecdysone, from plant extracts to clinical use: therapeutic potential for the treatment of neuromuscular, cardio-metabolic and respiratory diseases. Biomedicines. 2021;9(5):492. doi: 10.3390/biomedicines9050492 33947076 PMC8146789

[12] Lafont R, Balducci C, Dinan L. Ecdysteroids. Encyclopedia. 2021;1(4):1267–1302. doi: 10.3390/encyclopedia1040096

[13] Gorelick-Feldman J, Cohick W, Raskin I. Ecdysteroids elicit a rapid Ca2+ flux leading to akt activation and increased protein synthesis in skeletal muscle cells. Steroids. 2010;75(10):632–637. doi: 10.1016/j.steroids.2010.03.008 20363237 PMC3815456

[14] Parr MK, Zhao P, Haupt O, et al. Estrogen receptor beta is involved in skeletal muscle hypertrophy induced by the phytoecdysteroid ecdysterone. Mol Nutr Food Res. 2014;58(9):1861–1872. doi: 10.1002/mnfr.201300806 24974955

[15] Lafont R, Serova M, Didry-Barca B, et al. 20-hydroxyecdysone activates the protective arm of the RAAS via the MAS receptor. J Mol Endocrinol. 2021;68(2):77–87. doi: 10.1530/JME-21-0033 34825653

[16] Sláma K, Koudela K, Tenora J, et al. Insect hormones in vertebrates: anabolic effects of 20-hydroxyecdysone in Japanese quail. Springer Nat Link. 1996;52(7):702–706. doi: 10.1007/BF01925578 8698114

[17] Krátky F, Opletal L, Hejhalek J, et al. Effect of 20-hydroxyecdysone on the protein synthesis of pigs. Zivocisna Vyroba. 42(1997):445–451.

[18] Stopka P, Stancl J, Slama K. Effect of insect hormone, 20-hydroxyecdysone on growth and reproduction in mice. Acta Societatis Zoologicae Bohemicae. 63(1999):367–378.

[19] Syrov VN. Comparative experimental investigation of the anabolic activity of phytoecdysteroids and steranabols. Pharm Chem J. 2000;34(4):193–197. doi: 10.1007/BF02524596

[20] Tóth N, Szabó A, Kacsala P, et al. 20-hydroxyecdysone increases fiber size in a muscle-specific fashion in rat. Phytomedicine. 2008;15(9):691–698. doi: 10.1016/j.phymed.2008.04.015 18585021

[21] Kostov T, Diel P, Isenmann E. Examination of the anabolic activity and mechanisms of action of the combination of diosgenin and ecdysterone in C2C12 myotubes. Toxicol Lett. 2024;401:181–189. doi: 10.1016/j.toxlet.2024.10.005 39395682

[22] Kumpun S, Girault J-P, Dinan L, et al. The metabolism of 20-hydroxyecdysone in mice: relevance to pharmacological effects and gene switch applications of ecdysteroids. J Steroid Biochem Mol Biol. 2011;126(1–2):1–9. doi: 10.1016/j.jsbmb.2011.03.016 21439380

[23] Dinan L, Balducci C, Guibout L, et al. Ecdysteroid metabolism in mammals: the fate of ingested 20-hydroxyecdysone in mice and rats. J Steroid Biochem Mol Biol. 2021;212:105896. doi: 10.1016/j.jsbmb.2021.105896 33819630

[24] Pérez-Piñero S, Ávila-Gandía V, Rubio Arias JA, et al. A 12-week randomized double-blind placebo-controlled clinical trial, evaluating the effect of supplementation with a spinach extract on skeletal muscle Fitness in adults older than 50 years of age. Nutrients. 2021;13(12):4373. doi: 10.3390/nu13124373 34959924 PMC8706266

[25] Wilborn CD, Taylor LW, Campbell BI, et al. Effects of methoxyisoflavone, ecdysterone, and sulfo-polysaccharide supplementation on training adaptations in resistance-trained males. J Int Soc Sports Nutr. 2006;3(2):19–27. doi: 10.1186/1550-2783-3-2-19 18500969 PMC2129166

[26] Dioh W, Tourette C, Del Signore S, et al. A phase 1 study for safety and pharmacokinetics of BIO101 (20-hydroxyecdysone) in healthy young and older adults. J Cachexia Sarcopenia Muscle. 2023;14(3):1259–1273. doi: 10.1002/jcsm.13195 37057316 PMC10235879

[27] Lobo SM, Plantefève G, Nair G, et al. Efficacy of oral 20-hydroxyecdysone (BIO101), a MAS receptor activator, in adults with severe COVID-19 (COVA): a randomized, placebo-controlled, phase 2/3 trial. eClinicalmedicine. 2024;68:102383. doi: 10.1016/j.eclinm.2023.102383 38545090 PMC10965409

[28] Fielding RA, Dao MM, Cannon K, et al. BIO101 in Sarcopenic seniors at risk of mobility disability: results of a double-blind randomised interventional phase 2b trial. J Cachexia Sarcopenia Muscle. 2025;16(2):e13750. doi: 10.1002/jcsm.13750 40026058 PMC11873539

[29] Ambrosio G, Wirth D, Joseph JF, et al. How reliable is dietary supplement labelling?—experiences from the analysis of ecdysterone supplements. J Pharm Biomed Anal. 2020;177:112877. doi: 10.1016/j.jpba.2019.112877 31568967

[30] Bundesamt für verbraucherschutz und lebensmittelsicherheit, BVL. Nahrungsergänzungsmittel vs. Arzneimittel. [cited 2024 Oct 25]. Available from: https://www.bvl.bund.de/DE/Arbeitsbereiche/01_Lebensmittel/03_Verbraucher/04_NEM/01_NEM_Arzneimittel/NEM_Arzneimittel_node.html

[31] Outram S, Stewart B. Doping through supplement use: a review of the available empirical data. Int J Sport Nutr Exerc Metab. 2015;25(1):54–59. doi: 10.1123/ijsnem.2013-0174 25722470

[32] Geyer H, Parr MK, Koehler K, et al. Nutritional supplements cross-contaminated and faked with doping substances. J Mass Spectrom. 2008;43(7):892–902. doi: 10.1002/jms.1452 18563865

[33] Geyer H, Parr MK, Mareck U, et al. Analysis of non-hormonal nutritional supplements for anabolic-androgenic steroids - results of an international study. Int J Sports Med. 2004;25:124–129. doi: 10.1055/s-2004-819955 14986195

[34] Pálinkás Z, Békési D, Utczás M. Quantitation of ecdysterone and targeted analysis of WADA-Prohibited anabolic androgen steroids, hormones, and metabolic modulators in ecdysterone-containing dietary supplements. Separations. 2023;10(4):242. doi: 10.3390/separations10040242

[35] WADA. *The WADA 2020 monitoring program*. 2020 [cited 2024 Sep 5]. Available from: https://www.wada-ama.org/sites/default/files/wada_2020_english_monitoring_program_.pdf

[36] Grebenok RJ, Adler JH. Ecdysteroid distribution during development of spinach. Phytochemistry. 1991;30(9):2905–2910. doi: 10.1016/S0031-9422(00)98222-0

[37] Graf BL, Rojo LE, Delatorre-Herrera J, et al. Phytoecdysteroids and flavonoid glycosides among Chilean and commercial sources of Chenopodium quinoa: variation and correlation to physico-chemical characteristics. J Sci Food Agric. 2016;96(2):633–643. doi: 10.1002/jsfa.7134 25683633 PMC4534356

[38] Grebenok RJ, Adler JH. Ecdysteroid biosynthesis during the ontogeny of spinach leaves. Phytochemistry. 1993;33(2):341–347. doi: 10.1016/0031-9422(93)85514-r

[39] Isenmann E, Yuliandra T, Touvleliou K, et al. Quinoa as phytopharmaceutical? Urinary elimination of ecdysterone after consumption of quinoa alone and in combination with spinach. Archiv der Pharmazie. 2024;357(6):e2300689. doi: 10.1002/ardp.202300689 38400693

[40] Ambrosio G, Yuliandra T, Wuest B, et al. Urinary elimination of ecdysterone and its metabolites following a single-dose administration in humans. Metabolites. 2021;11(6):366. doi: 10.3390/metabo11060366 34207569 PMC8227119

[41] Yuliandra T, Touvleliou K, de La Torre X, et al. Urinary excretion of ecdysterone and its metabolites following spinach consumption. Mol Nutr Food Res. 2023;67(14):e2200518. doi: 10.1002/mnfr.202200518 37161586

[42] Jesus M, Martins APJ, Gallardo E, et al. Diosgenin: recent highlights on pharmacology and analytical methodology. J Anal Methods Chem. 2016;2016:1–16. doi: 10.1155/2016/4156293 PMC5225340 28116217

[43] Chen Y, Tang Y-M, Yu S-L, et al. Advances in the pharmacological activities and mechanisms of diosgenin. Chin J Nat Med. 2015;13(8):578–587. doi: 10.1016/S1875-5364(15)30053-4 26253490

[44] Fuller S, Stephens JM. Diosgenin, 4-hydroxyisoleucine, and fiber from fenugreek: mechanisms of actions and potential effects on metabolic syndrome. Adv Nutr. 2015;6(2):189–197. doi: 10.3945/an.114.007807 25770257 PMC4352177

[45] Parama D, Boruah M, Yachna K, et al. Diosgenin, a steroidal saponin, and its analogs: effective therapies against different chronic diseases. Life Sci. 2020;260:118182. doi: 10.1016/j.lfs.2020.118182 32781063

[46] Semwal P, Painuli S, Abu-Izneid T, et al. Diosgenin: an updated pharmacological review and therapeutic perspectives. Oxid Med Cell Longev. 2022;2022(1):1035441. doi: 10.1155/2022/1035441 35677108 PMC9168095

[47] Rao A, Clayton P, Briskey D. Libifem® (Trigonella foenum-graecum) in conjunction with exercise on muscle strength, power, endurance, and body composition in females aged between 25 and 45 years. Front Sports Act Living. 2023;5:1207013. doi: 10.3389/fspor.2023.1207013 37637219 PMC10450923

[48] Fahey TD. The steroid alternative handbook : understanding anabolic steroids and drug-free scientific natural alternatives/by Thomas D. Fahey with Bob Fritz;. San Jose, CA: Sport Science Publications; 1991. ISBN 1878920006.

[49] Santos Junior ERT, Salles BFD, Dias I, et al. Classification and determination model of resistance training status. Strength Cond J. 2021;43(5):77–86. doi: 10.1519/SSC.0000000000000627

[50] McKay AKA, Stellingwerff T, Smith ES, et al. Defining training and performance caliber: a participant classification framework. Int J Sports Physiol Perform. 2022;17(2):317–331. doi: 10.1123/ijspp.2021-0451 34965513

[51] Ioannidou P, Dóró Z, Schalla J, et al. Analysis of combinatory effects of free weight resistance training and a high-protein diet on body composition and strength capacity in postmenopausal women - a 12-week randomized controlled trial. J Nutr Health Aging. 2024;28(10):100349. doi: 10.1016/j.jnha.2024.10034939232439

[52] Gavanda S, Geisler S, Quitmann OJ, et al. Three weeks of detraining does not decrease muscle thickness, strength or sport performance in adolescent athletes. Int J Exerc Sci. 2020;13:633–644. 32509134 10.70252/LUXA7451 PMC7241623

[53] Lukaski HC, Bolonchuk WW, Hall CB, et al. Validation of tetrapolar bioelectrical impedance method to assess human body composition. J Appl Physiol. 1986;60(4):1327–1332. doi: 10.1152/jappl.1986.60.4.1327 3700310

[54] Gavanda S, Geisler S, Quittmann OJ, et al. The effect of block versus daily undulating periodization on strength and performance in adolescent football players. Int J Sports Physiol Perform. 2019;14(6):814–821. doi: 10.1123/ijspp.2018-0609 30569761

[55] Isenmann E, Kaluza D, Havers T, et al. Resistance training alters body composition in middle-aged women depending on menopause - a 20-week control trial. BMC Women’s Health. 2023;23. doi: 10.1186/s12905-023-02671-y PMC10559623 37803287

[56] Yasuda T, Fujita S, Ogasawara R, et al. Effects of low-intensity bench press training with restricted arm muscle blood flow on chest muscle hypertrophy: a pilot study. Clin Physiol Funct Imaging. 2010;30(5):338–343. doi: 10.1111/j.1475-097X.2010.00949.x 20618358

[57] Abe T, DeHoyos DV, Pollock ML, et al. Time course for strength and muscle thickness changes following upper and lower body resistance training in men and women. Eur J Appl Physiol. 2000;81(3):174–180. doi: 10.1007/s004210050027 10638374

[58] Enes A, Oneda G, Leonel DF, et al. The effects of squat variations on strength and quadriceps hypertrophy adaptations in recreationally trained females. Eur J Sport Sci. 2024;24(1):6–15. doi: 10.1002/ejsc.12042

[59] Haff G; Triplett NT, editors. Essentials of strength training and conditioning. Fourth ed. Human Kinetics: Champaign, US; 2016. ISBN 9781718210868.

[60] Koo TK, Li MY. A guideline of selecting and reporting intraclass correlation coefficients for reliability research. J Chiropr Med. 2016;15(2):155–163. doi: 10.1016/j.jcm.2016.02.012 27330520 PMC4913118

[61] Helms ER, Kwan K, Sousa CA, et al. Methods for regulating and monitoring resistance training. J Hum Kinet. 2020;74(1):23–42. doi: 10.2478/hukin-2020-0011 33312273 PMC7706636

[62] Helms ER, Byrnes RK, Cooke DM, et al. RPE vs. percentage 1RM loading in periodized programs matched for sets and repetitions. Front Physiol. 2018;9:247. doi: 10.3389/fphys.2018.00247 29628895 PMC5877330

[63] Baum Martinez I, Peters B, Schwarz J, et al. Validation of a smartphone application for the assessment of dietary compliance in an intermittent fasting trial. Nutrients. 2022;14(18):3697. doi: 10.3390/nu14183697 36145073 PMC9506329

[64] Haaf TT, Weijs PJM, Alemany M. Resting energy expenditure prediction in recreational athletes of 18–35 years: confirmation of cunningham equation and an improved weight-based alternative. PLOS ONE. 2014;9(10):e108460. doi: 10.1371/journal.pone.0108460 25275434 PMC4183531

[65] Morton RW, Murphy KT, McKellar SR, et al. A systematic review, meta-analysis and meta-regression of the effect of protein supplementation on resistance training-induced gains in muscle mass and strength in healthy adults. Br J Sports Med. 2018;52(6):376–384. doi: 10.1136/bjsports-2017-097608 28698222 PMC5867436

[66] Jiang L, Piribauer M, Kostov T, et al. Testing anabolic activity, potency and mechanisms of action of the phyto-derived beta 2 agonist higenamine. Toxicol Lett. 2023;385:21–28. doi: 10.1016/j.toxlet.2023.08.007 37598871

[67] Piribauer M, Jiang L, Kostov T, et al. Combinatory in vitro effects of the β2-agonists salbutamol and formoterol in skeletal muscle cells. Toxicol Lett. 2023;378:10–18. doi: 10.1016/j.toxlet.2023.02.007 36822333

[68] Zheng W, Hemker ML, Xie M, et al. Anabolic activity of a soy extract and three major isoflavones in C2C12 myotubes. Planta Med. 2018;84(14):1022–1029. doi: 10.1055/a-0598-4812 29649842

[69] The R Core Team R. A language and environment for statistical computing: reference index. [cited 2024 Sep 28]. Available from: https://cran.r-project.org/doc/manuals/r-release/fullrefman.pdf

[70] Bates D, Mächler M, Bolker B, et al. Fitting linear mixed-effects models using lme4. J Stat Soft. 2015;67(1). doi: 10.18637/jss.v067.i01

[71] Kuznetsova A, Bruun Brockhoff P, Haubo Bojesen Christensen R. CRAN: Contributed Packages. 2013.

[72] Lenth RV. CRAN: contributed packages. 2017.

[73] Cohen J. Statistical power analysis for the behavioral sciences;. New York: Routledge; 2013. ISBN 9781134742707.

[74] Wickham H, Chang W, Henry L, et al. Teun van den brand. ggplot2: elegant graphics for data analysis. Available from: https://ggplot2.tidyverse.org

[75] Benito PJ, Cupeiro R, Ramos-Campo DJ, et al. A systematic review with meta-analysis of the effect of resistance training on whole-body muscle growth in healthy adult males. Int J Environ Res Public Health. 2020;17(4):1285. doi: 10.3390/ijerph17041285 32079265 PMC7068252

[76] Cohen PA, Sharfstein J, Kamugisha A, et al. Analysis of ingredients of supplements in the National institutes of health supplement database marketed as containing a novel alternative to anabolic steroids. JAMA Netw Open. 2020;3(4):e202818. doi: 10.1001/jamanetworkopen.2020.2818 32293681 PMC7160690

[77] Crawford C, Avula B, Lindsey AT, et al. Label accuracy of weight loss dietary supplements marketed online with military discounts. JAMA Netw Open. 2024;7(5):e249131. doi: 10.1001/jamanetworkopen.2024.9131 38691359 PMC11063798

[78] Crawford C, Avula B, Lindsey AT, et al. Analysis of select dietary supplement products marketed to support or boost the immune system. JAMA Netw Open. 2022;5(8):e2226040. doi: 10.1001/jamanetworkopen.2022.26040 35947382 PMC9366544

[79] Crawford C, Walter AR, Avula B, et al. Relative safety and quality of various dietary supplement products U.S. Service members ask about. Clin Toxicol (phila). 2022;60(6):737–744. doi: 10.1080/15563650.2022.2036751 35156875

[80] Geyer H, Bredehöft M, Mareck U, et al. High doses of the anabolic steroid metandienone found in dietary supplements. Eur J Sport Sci. 2003;3(1):1–5. doi: 10.1080/17461390300073102

[81] Schänzer W, Thevis M, Geyer H, Mareck U, editors. Proceedings of the Manfred Donike workshop, 35th Cologne workshop on dope analysis: 5th to 10th March 2017. 1 Auflage ed. Köln: Sportverlag Strauß; 2018. ISBN 3868840435.

[82] U.S. Food & Drug Administration. Dietary supplements. [cited 2024 Oct 25]. Available from: https://www.fda.gov/food/dietary-supplements

[83] Navarro VJ, Khan I, Björnsson E, et al. Liver injury from herbal and dietary supplements. Hepatology. 2017;65(1):363–373. doi: 10.1002/hep.28813 27677775 PMC5502701

[84] Kozhuharov VR, Ivanov K, Ivanova S, et al. Dietary supplements as source of unintentional doping. Biomed Res Int. 2022;2022(1):8387271. doi: 10.1155/2022/8387271 35496041 PMC9054437

[85] Bajkacz S, Rusin K, Wolny A, et al. Highly efficient extraction procedures based on natural deep eutectic solvents or ionic liquids for determination of 20-hydroxyecdysone in spinach. Molecules. 2020;25(20):4736. doi: 10.3390/molecules25204736 33076445 PMC7587567

[86] Cheng DM, Yousef GG, Lila MA. Variation in phytoecdysteroid accumulation in seeds and shoots of Spinacia oleracea L. Accessions. Horts. 2010;45(11):1634–1638. doi: 10.21273/HORTSCI.45.11.1634

[87] Bakrim A, Maria A, Sayah F, et al. Ecdysteroids in spinach (Spinacia oleracea L.): biosynthesis, transport and regulation of levels. Plant Physiol Biochem. 2008;46(10):844–854. doi: 10.1016/j.plaphy.2008.06.002 18653353

[88] Dinan L, Savchenko T, Whiting P. On the distribution of phytoecdysteroids in plants. Cell Mol Life Sci. 2001;58(8):1121–1132. doi: 10.1007/PL00000926 11529504 PMC11337386

[89] Fang X, Szołtysik R, Tang J, et al. Efficient extraction and sensitive HPLC-MS/MS quantification of selected ecdysteroids in plants. J Food Composition Anal. 2022;110:104580. doi: 10.1016/j.jfca.2022.104580

[90] Gorelick J, Iraqi RH, Bernstein N. Ecdysteroid content and therapeutic activity in elicited spinach accessions. Plants (Basel). 2020;9(6):727. doi: 10.3390/plants9060727 32526841 PMC7356866

[91] Hartgens F, Kuipers H. Effects of androgenic-anabolic steroids in athletes. Sports Med. 2004;34:513–554. doi: 10.2165/00007256-200434080-00003 15248788

[92] Rao AJ, Mallard AR, Grant R. Testofen® (Fenugreek extract) increases strength and muscle mass compared to placebo in response to calisthenics. A randomized control trial. Transl Sports Med. 2020;3(4):374–380. doi: 10.1002/tsm2.153

